# Combination of cisplatin and bromelain exerts synergistic cytotoxic effects against breast cancer cell line MDA-MB-231 in vitro

**DOI:** 10.1186/s13020-016-0118-5

**Published:** 2016-11-15

**Authors:** Ahmad Zaim Mat Pauzi, Swee Keong Yeap, Nadiah Abu, Kian Lam Lim, Abdul Rahman Omar, Suraini Abdul Aziz, Adam Leow Thean Chow, Tamilselvan Subramani, Soon Guan Tan, Noorjahan Banu Alitheen

**Affiliations:** 1 Department of Cell and Molecular Biology, Faculty of Biotechnology and Biomolecular Sciences, Universiti Putra Malaysia, 43400 Serdang, Selangor Malaysia; 2 Institute of Bioscience, Universiti Putra Malaysia, Serdang, Malaysia; 3 Faculty of Medicine and Health Sciences, Universiti Tunku Abdul Rahman, Sungai Long Campus, Jalan Sungai Long, Bandar Sungai Long, Cheras, 43000 Kajang, Selangor Malaysia; 4 Department of Bioprocess Technology, Faculty of Biotechnology and Biomolecular Sciences, Universiti Putra Malaysia, Serdang, Selangor Malaysia

## Abstract

**Background:**

Bromelain, which is a cysteine endopeptidase commonly found in pineapple stems, has been investigated as a potential anti-cancer agent for the treatment of breast cancer. However, information pertaining to the effects of combining bromelain with existing chemotherapeutic drugs remains scarce. This study aimed to investigate the possible synergistic cytotoxic effects of using bromelain in combination with cisplatin on MDA-MB-231 human breast cancer cells.

**Method:**

MDA-MB-231 cells were treated with different concentrations (0.24–9.5 µM) of bromelain or cisplatin alone, as well as four different combinations of these two agents to assess their individual and combination effects after 24 and 48 h. Cell viability was analyzed using an MTT assay. The induction of apoptosis was assessed using cell cycle analysis and an Annexin V-FITC assay. The role of the mitochondrial membrane potential in the apoptotic process was assessed using a JC-1 staining assay. Apoptotic protein levels were assessed by western blot analysis and proteome profiling using an antibody array kit.

**Results:**

Single-agent treatment with cisplatin or bromelain led to dose- and time-dependent decreases in the viability of the MDA-MB-231 cells at 24 and 48 h. Furthermore, most of the combinations evaluated in this study displayed synergistic effects against MDA-MB-231 cells at 48 h, with combination 1 (bromelain 2 µM + cisplatin 1.5 µM) exhibiting the greatest synergistic effect (*P* = 0.000). The results of subsequent assays indicated that combination 1 treatment induced apoptosis via mitochondria-mediated pathway. Combination 1 also resulted in significant decreases in the levels of several apoptotic proteins such as Bcl-x and HSP70, compared with bromelain (*P* = 0.002 and 0.000, respectively) or cisplatin (*P* = 0.000 and 0.001, respectively) single treatment. Notably, MDA-MB-231 cells treated with combination 1 showed increased levels of the pro-apoptotic proteins Bax compared with those treated with bromelain (*P* = 0.000) or cisplatin single treatment (*P* = 0.043).

**Conclusion:**

Bromelain in combination with cisplatin synergistically enhanced the induction of apoptosis in MDA-MB-231 cells.

## Background

Breast cancer is the most frequently diagnosed invasive non-skin malignancy and the leading cause of cancer-related deaths in women throughout the world [1]. In Asia, the incidence and mortality rates of breast cancer have been steadily rising for several years, with unhealthy diet, physical inactivity and obesity being identified as the main contributing factors [2]. Although there are many different strategies available for the clinical treatment of breast cancer, the effectiveness of these approaches can be limited by the occurrence of adverse side effects and the development of drug resistance.

cis-Diamminedichloroplatinum (CDDP), which is more commonly known as cisplatin, is one of the most effective anti-cancer agents currently used in clinical practice, with pronounced activity against various cancers, including breast cancer [3, 4]. Cisplatin interacts with DNA and interferes with the mechanisms responsible for its transcription and replication [5, 6]. Consequently, cisplatin treatment is associated with a number of serious side effects such as nephrotoxicity, myelosuppression, ototoxicity, anaphylactic reactions, peripheral neuropathies and hypomagnesemia [7–9]. Furthermore, the clinical application of cisplatin is limited by the development of resistance mechanisms in the cancer cells [10–12]. The combination of cisplatin with other anti-cancer agents that operate via a different mode of action could therefore be used as an effective strategy to impede the growth of human cancer cells that develop resistance to cisplatin. This strategy could also minimize the severity of the side effects associated with the individual agents, whilst maintaining or even enhancing the effectiveness of the treatment process.

Pineapple (*Ananascomosus* L.) has been used to treat a wide range of diseases in several different countries, including Thailand, Malaysia, Taiwan and China, as well as the state of Hawaii [13]. Pineapple plants are commonly used in folk medicine, especially their crown leaves, which are used to treat open wounds and inflammation. The results of a previous study demonstrated that pineapple crown leaf extract exhibited several interesting biological properties, including antimicrobial, anti-edema and anti-inflammatory activities [14].Pineapple stems have also been reported to exhibit a broad range of promising pharmacological properties. Stem bromelain is a cysteine endopeptidase, which is commonly found at a high concentration in the crude extract of pineapple stems (*Ananascomosus* L.) [15]. The results of several in vitro and in vivo studies [16–21] have demonstrated that bromelain exhibited various beneficial therapeutic effects, including anti-tumor activity. These results therefore support the potential application of stem bromelain as a therapeutic agent for the treatment of cancer. Moreover, bromelain exhibits good stability over a wide range of pH values [22, 23] and is readily adsorbed in the human intestinal tract in its functional active form when it is consumed in high concentrations (up to 12 g/day). Taken together with the fact that its consumption does not lead to any major side effects, these results further highlight the potential of bromelain as an anti-cancer agent [24, 25].

The study aimed to investigate the possible synergistic cytotoxic effects of using bromelain in combination with cisplatin for the treatment of MDA-MB-231 human breast cancer cells.

## Methods

### Chemicals and reagents

Unless specified otherwise, all of the chemicals used in this study, including bromelain and cisplatin, were obtained from Sigma Aldrich (St Louis, MO, USA). Stock solutions of bromelain in water were freshly prepared prior to each experiment using deionized water. The resulting aqueous solutions were filtered (0.2 µm) prior to being used in the experiments. A stock solution of cisplatin was prepared in the dark using deionized water containing 0.9% (w/w) sodium chloride. The resulting stock solution was stored at 4 °C in the absence of light prior to being used.

### Cell cultures

The MDA-MB-231 cells used in this study obtained from the American Type Culture Collection (Rockville, MD, USA). The cells were cultured in Roswell Park Memorial Institute medium enriched with 10% fetal bovine serum and 100 units/mL penicillin–streptomycin antibiotic at 37 °C under a humidified atmosphere containing 5% CO_2_.

### MTT assay

Cell growth inhibition was determined using a colorimetric MTT assay. The assay was conducted in a 96-well plate with a cell density of 8 × 10^3^ cells per well with an incubation period of 24 h. The medium was subsequently removed and replaced with fresh medium containing the test compound, followed by an incubation period of 24 or 48 h. The cells were then incubated with MTT solution (0.5 mg/mL) for 4 h, and the resulting formazan precipitate was dissolved in 170 µL of DMSO. The absorbance of each well was then measured at 570 nm using a microplate spectrophotometer (Bio-Tek Instruments, Winooski, VT, USA). The percentage of cell survival was calculated using the following formula: percentage (%) cell survival = [(mean absorbency in test wells)/(mean absorbency in control wells)] × 100. These experiments were conducted in triplicate. We then constructed a graph of the percentage cell viability against the concentration of the test compound. The resulting graph was used to determine the IC10, IC20, IC30, IC40 and IC50 values of bromelain and cisplatin for the single treatment of the MDA-MB-231 cells.

We also conducted a series of MTT assays using four different combinations of bromelain and cisplatin (*i.e.*, IC40 bromelain + IC10 cisplatin, IC30 bromelain + IC20 cisplatin, IC20 bromelain + IC30 cisplatin and IC10 bromelain + IC40 cisplatin) with concentrations in the range of 0.24–9.5 µM. All of these assays were conducted in a 96-well plate with a cell density of 8 × 10^3^ cells per well with an incubation period of 24 h. The medium was subsequently removed and replaced with fresh medium containing the test compound, followed by an incubation period of 48 h. The cells were then incubated with MTT solution (0.5 mg/mL) for 4 h, and the resulting formazan precipitate was dissolved in 170 µL of DMSO. The absorbance of each well was then measured at 570 nm using a microplate spectrophotometer (Bio-Tek Instruments). All of these experiments were conducted independently in triplicate.

### Annexin V-FITC assay

The cells were seeded into a 6-well plate and incubated for 24 h at 37 °C under a humidified atmosphere containing 5% CO_2_. The medium in each well was subsequently replaced with fresh medium containing different concentrations of the test compounds. After an incubation period of 24 or 48 h, all of the detached/dead and viable cells were collected. The cells were then washed and resuspended with PBS. The harvested cells were stained with Annexin V for 30 min before being treated with PI and analyzed by flow cytometry using a FACScan system (Becton–Dickinson and Company, San Jose, CA, USA) equipped with version 3.3 of the CellQuest software (Becton–Dickinson and Company). This assay was conducted according to the manufacturer’s protocol (BD PharmingenAnnexin V-FITC Apoptosis Detection Kit 1).

### Measurement of mitochondrial membrane potential (JC-1 staining assay)

The cells were seeded into a 6-well plate and incubated for 24 h at 37 °C under a humidified atmosphere containing 5% CO_2_. The medium in each well was then replaced with fresh medium containing different concentrations of the test compounds. After an incubation period of 48 h, all the detached/dead and viable cells were harvested, washed with PBS and incubated with culture medium containing JC-1 for 30 min at 37 °C in the absence of light. The cells were then washed twice with PBS, resuspended in 500 µL of PBS and immediately analyzed by flow cytometry using a FACScan system (Becton–Dickinson and Company) equipped version 3.3 of the CellQuest software.

### Cell cycle analysis

The cells were seeded into a 6-well plate and incubated for 24 h at 37 °C under a humidified atmosphere containing 5% CO_2_. The medium in each well was then replaced with fresh medium containing different concentrations of the test compounds. After an incubation period of 24 or 48 h, all of the detached/dead and viable cells were collected, washed with cold PBS and resuspended in 50 mL of cold PBS before being treated with 450 µL of cold ethanol. The cells were then incubated for 24 h at 4 °C. At the end of the incubation period, the cells were centrifuged (Model 5804 R, Eppendorf, Hamburg, Germany) at 200×*g* for 5 min and the resulting pellet was washed with cold PBS and resuspended in 500 µL of PBS. The cells were then incubated with 5 µL of RNase (20 μg/mL final concentration) for 30 min before being incubated with PI (50 µg/mL) on ice for 1 h in the dark. The distribution of cells was then immediately analyzed by flow cytometry using a FACScan system (Becton–Dickinson and Company) equipped with version 3.3 of the CellQuest software.

### Proteome Profiler™: human apoptosis array

The cells were seeded in a 6-well plate and incubated for 24 h at 37 °C under a humidified atmosphere containing 5% CO_2_. The medium in each well was then replaced with fresh medium containing different concentrations of the test compounds. After an incubation period of 48 h, the cells were washed with PBS and lysed. All of the immunodetection steps were performed using a Proteome Profiler Human Apoptosis Array Kit (R&D Systems, Minneapolis, MN, USA) in accordance with the manufacturer’s instructions. Briefly, the array was washed and incubated with a mixture of biotinylated detection antibodies. Streptavidin-HRP and chemiluminescent detection reagents were used, and a signal corresponding to the amount of protein bound was produced on each capture spot. After incubation, the membranes were developed using enhanced chemiluminescence reagents and immediately viewed and analyzed using a ChemiDoc XRS + system (Bio-Rad, Hercules, CA, USA). Protein expression was normalized to a positive control, which was present in each membrane.

### Western blot

The cells were seeded into a 6-well plate and incubated for 24 h at 37 °C under a humidified atmosphere containing 5% CO_2_. The medium in each well was then replaced with fresh medium containing different concentrations of the test compound. Following an incubation period of 24 or 48 h, the cells were washed with PBS and lysed in RIPA lysis buffer [50 mM Hepes (pH 7.5), 150 mM NaCl, 1% deoxycholate, 1% NP-40, 0.1% sodium dodecyl sulfate (SDS)] containing protease inhibitors (Thermo Fisher Scientific, Waltham, MA, USA). The extracted proteins (20–60 µg) were separated by electrophoresis on SDS–polyacrylamide gels, transferred to nitrocellulose membranes (Bio-Rad) and probed with respective primary antibodies against Beta-actin, Bax and Bcl-2 (Abcam, Cambridge, Massachusetts, USA). After being incubated with the corresponding secondary antibodies (Abcam, the immunoreacted proteins were detected using a chemiluminescence system (ECL Western blot substrate; Abcam, Cambridge, UK).The bands obtained were quantitated using the ImageJ software (Bio Techniques, New York, NY, USA).

### Statistical analysis

All of the experiments described in this study were repeated independently for at least three times (n = 3) and measured in triplicate, unless specified otherwise. The results have been reported as the corresponding mean values ± standard deviation (SD). Statistical data for MTT assay, Annexin-V/FitC assay, JC-1 staining assay and cell cycle analysis was analyzed by the Student’s *t* test and one-way analysis of variance (ANOVA), followed by Tukey’s multiple comparison post hoc test. Statistical analyses were performed using version 19 of the SPSS software for Windows (IBM SPSS, Chicago, IL, USA). Differences between the experimental groups were considered significant for *P* values of less than 0.05. Data analyses to determine the CI value of the combination treatment used were performed using the CompuSyn software (Combo SynInc, City, State, USA). CI < 1 indicates synergism, CI = 1 indicates additive effect, and CI > 1 indicates antagonism.

## Results

### Inhibitory effects of cisplatin and bromelain (single-agent and combined treatment) on the growth of MDA-MB-231 cells

**Fig. 1:**
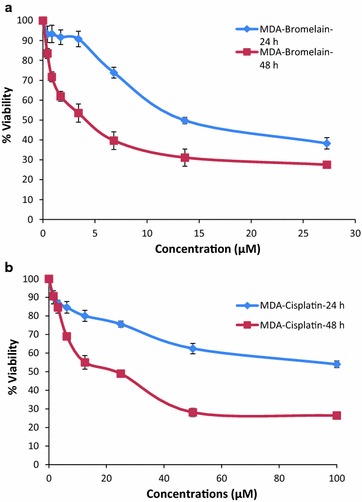
Dose- and time-dependent inhibition of viability in MDA-MB-231 cells by bromelain and cisplatin single treatment. The figure shown in **a** is the MTT assay results for bromelain at 24 and 48 h, with IC50 value for bromelain at 48 h is 4.10 µM. The figure shown in **b** is the MTT assay results for cisplatin at 24 and 48 h, with IC50 value for cisplatin at 48 h is 23.0 µM. The results are expressed as mean ± SD of three separate experiments

The proliferation of MDA-MB-231 cells was assessed using an MTT assay. The assay was conducted in the presence of various concentrations of cisplatin or bromelain, as well as different combinations of these two agents over 24 and 48 h to explore their effects on the MDA-MB-231 cells. The treatment of the cells with cisplatin or bromelain alone resulted in a dose-dependent and a time-dependent decrease in the viability of the MDA-MB-231 cells at 24 and 48 h, with higher concentrations and longer treatment times resulting in lower levels of cell viability (Fig. 1).

These results were used to determine the concentrations of cisplatin and bromelain required to inhibit the growth of the MDA-MB-231 cells by 50, 40, 30, 20 and 10% (*i.e.*, the IC50, IC40, IC30, IC20 and IC10 values) at 48 h. Based on these values, we were able to combine these concentrations into four different combinations of cisplatin and bromelain, which were subsequently tested against MDA-MB-231 cells for 48 h. The results of these combination studies are shown in Table 1. Combination 1 (bromelain 2 µM + cisplatin 1.5 µM) induced the greatest reduction in cell viability compared to combination 2 (*P* = 0.000), combination 3 (*P* = 0.000) and combination 4 (*P* = 0.000), indicating that it exhibited the strongest inhibitory effect of the four combinations.

**Table 1:** Viability percentage of MDA-MB-231 at 48 h treated with bromelain and cisplatin combinations

Combination no.	Combination dosage (µM)		% viability ± SD
	Bromelain dosage (IC value)	Cisplatin dosage (IC value)	
Combination 1	2.0 µM (IC40)	1.5 µM (IC10)	50.24% ± 1.67
Combination 2	0.9 µM (IC30)	4.0 µM (IC20)	70.26% ± 2.66
Combination 3	0.5 µM (IC20)	5.9 µM (IC30)	61.30% ± 2.43
Combination 4	0.24 µM (IC10)	9.5 µM (IC40)	56.95% ± 3.61

Cytotoxicity was determined using the MTT cell viability assay. The results for MTT assay are expressed as mean ± SD of three separate experiments

### Synergistic effects of combinations of cisplatin and bromelain for the treatment of MDA-MB-231 cells

**Fig. 2:**
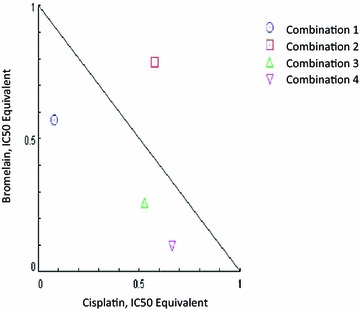
Normalized isobologram plot of bromelain/cisplatin combinations treatment on MDA-MB-231 cells at 48 h

The CI and DRI values, as well as the isobolograms for all of the bromelain and cisplatin combinations, are shown in Table 2. Only combinations 1, 3 and 4 gave CI values of less than 1, with combination 1 showing the lowest CI value of all of the combinations tested in the current study (CI 0.65). The results of the isobolograms were consistent with those of the CI values and revealed the presence of synergistic effects for combinations 1, 3 and 4 at 48 h (Fig. 2). The DRI values also supported the presence of synergistic effects, especially for combination 1, which had the highest DRI value for cisplatin. The DRI values showed that the IC50 dose of cisplatin could be reduced 13-fold when it was used in combination with bromelain in combination 1. These results indicated that combination 1 showed the best synergistic effects of all of the combinations tested in this study at 48 h. This combination was therefore selected for the subsequent experiments.

**Table 2:** Combination index (CI) and dose reduction index (DRI) values at 48 h for bromelain and cisplatin combinations. CI < 1 indicates synergistic effect, CI = 1 indicates additive effects and CI > 1 indicates antagonistic effect

Combination no.	Combination dosage (µM)		CI value	DRI (bromelain)	DRI (cisplatin)
	Bromelain dosage (IC value)	Cisplatin dosage (IC value)			
Combination 1	2.0 µM (IC40)	1.5 µM (IC10)	0.65	1.74	12.71
Combination 2	0.9 µM (IC30)	4.0 µM (IC20)	1.36	1.27	1.73
Combination 3	0.5 µM (IC20)	5.9 µM (IC30)	0.78	3.87	1.89
Combination 4	0.24 µM (IC10)	9.5 µM (IC40)	0.75	10.59	1.50

### Regulation of apoptosis and cell cycle progression in MDA-MB-231 cells by bromelain, cisplatin and their combined treatment

**Fig. 3:**
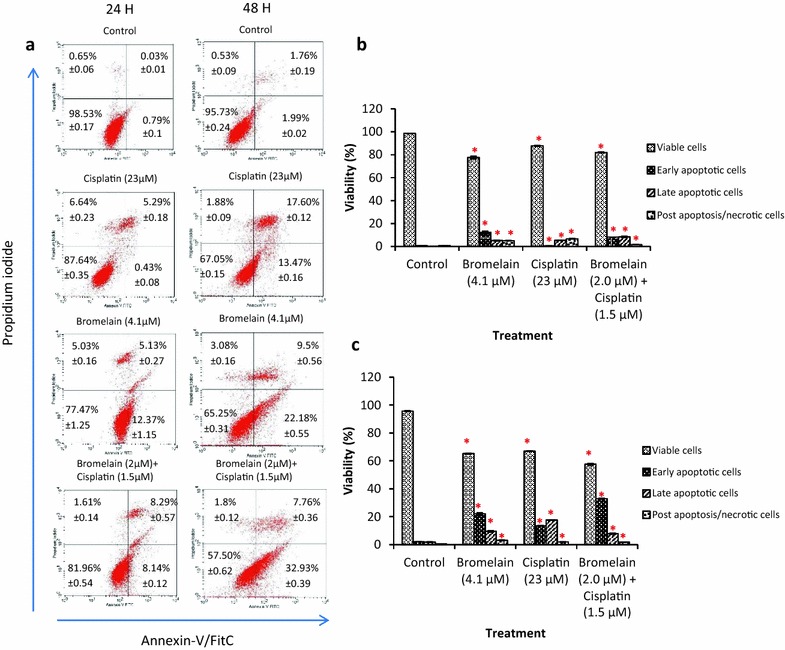
FITC-Annexin V/PI and propidium iodide staining of MDA-MB-231 cells treated with bromelain, cisplatin and bromelain/cisplatin combination for 24 and 48 h. The results are expressed as mean ± SD of three separate experiments. The figures shown in **a** are results from one of the three experiments conducted. The *x-axis* (Annexin-V/FITC) and *y-axis* (propidiumiodide) represent the intensity of Annexin-V/FITC and propidium iodide staining in cells, respectively. Shown in **b** and **c** are the analyzed results for 24 and 48 h, respectively. The numerical data are presented as mean ± SD (n = 3). *P < 0.05 vs. Control

**Fig. 4:**
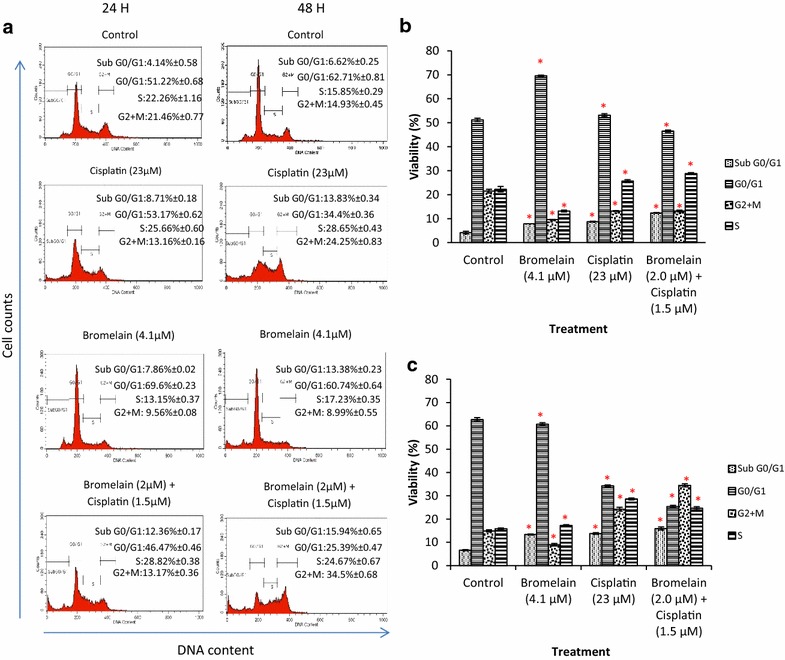
Effect of bromelain, cisplatin and bromelain/cisplatin combination treatment on MDA-MB-231 cell cycle after 24 and 48 h. The figures shown in **a** are the results from one of the three experiments conducted. Data shown represent relative numbers of cells (*Y-axis*) vs. DNA content (*X-axis*). Shown in **b** and **c** are the analyzed cell cycle results for 24 and 48 h respectively. The numerical data are presented as mean ± SD (n = 3). *P < 0.05 vs. Control

Annexin-V/PI and cell cycle analyses were performed on the MDA-MB-231 cells treated with combination 1 to determine whether the growth inhibitory effects of this treatment could be attributed to apoptosis or cell cycle arrest. The result of the Annexin-V/PI assay showed that the single-agent treatment of the MDA-MB-231 cells with bromelain (4.1 µM) or cisplatin (23 µM) caused 31.68 and 31.07% of the cells to enter apoptosis at 48 h, respectively (Fig. 3). However, the treatment of MDA-MB-231 cells with combination 1 led to a marked increase in the population of apoptotic cells observed at 48 h to 40.69%. These results correlated well with the fraction of cells observed in sub-G0/G1 phase by cell cycle analysis (Fig. 4). The treatment of the MDA-MB-231 cells with combination 1 led to a higher level of sub-G0/G1 arrest, with 15.94% of the cells being detected in the sub-G0/G1 phase, compared with values of 13.38 and 13.83% for single-agent treatment with bromelain (*P* = 0.000) and cisplatin (*P* = 0.000), respectively. Single-agent treatment with bromelain and cisplatin, as well as combination treatment with combination 1, induced apoptosis in MDA-MB-231 cells, with the former inducing higher levels of apoptosis than either of the single-agent treatments.

### Activation of apoptosis via an intrinsic (mitochondrial) pathway in MDA-MB-231 cells treated with a combination of bromelain and cisplatin

**Fig. 5:**
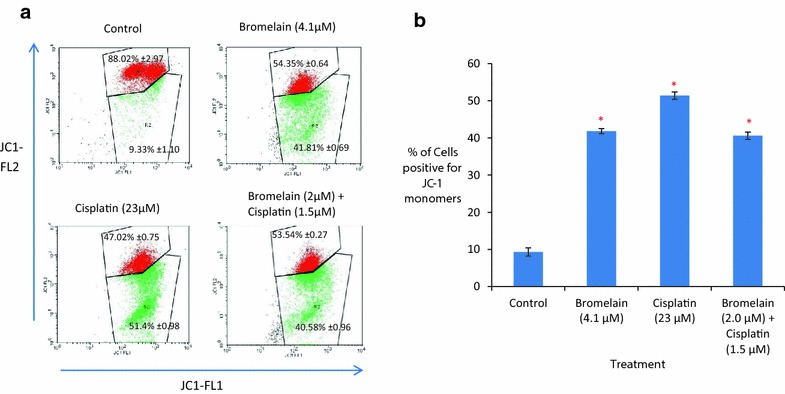
Effect of bromelain, cisplatin and bromelain/cisplatin combination after 48 h treatment duration on mitochondrial membrane potential in MDA-MB-231 cells as measured by JC-1 staining. The figures shown in **a** are the results from one of the three experiments conducted. Locations of R1 region (cells population with normal mitochondrial potential) and R2 region (cells population with reduced mitochondrial potential) are marked for *each plot*. Shown in **b** are the percentages of cells positive for JC-1 monomers for the treated and untreated MDA-MB-231 cells. The numerical data are presented as mean ± SD (n = 3). *P < 0.05 vs. Control

A JC-1 staining assay was used to measure changes in the mitochondrial membrane potentials of the MDA-MB-231 cells subjected to the single-agent and combination treatments. As shown in Fig. 5, the percentages of cells determined to be positive for JC-1 monomers following their single-agent treatment with bromelain (4.1 µM) and cisplatin (23 µM) for 48 h were 41.82% and 51.40%, respectively. For combination 1, the percentage of cells found to be positive for JC-1 monomers was 40.58%. These values were significantly higher than that found in the untreated control, where only 9.33% of the cells tested positive for JC-1 monomers (*P* = 0.000). The mitochondrial membrane potentials of the treated cells decreased, suggesting that the treatment of the cells with either of the single agents or combination 1 triggered apoptosis via the induction of the intrinsic (mitochondrial) pathway.

### Modulatory effects of bromelain and cisplatin alone, as well as a combination of these two agents on the expression levels of apoptosis-related proteins

**Fig. 6:**
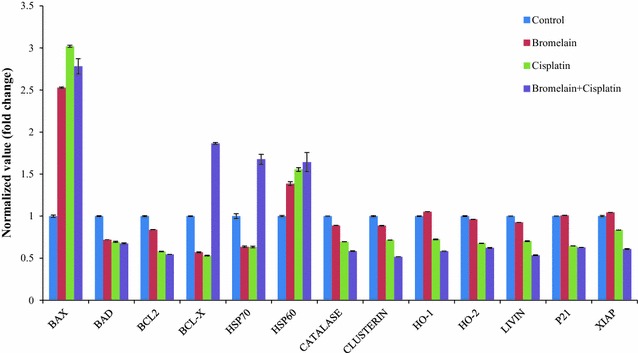
Alterations of apoptosis-related proteins by bromelain, cisplatin and bromelain/cisplatin combination treatment at 48 h. Shown here are the fold-change values for the pixel density of selected apoptotic proteins of treated cells to untreated cells (control) at 48 h, obtained using Proteome Profiler: human apoptosis array (R&D System). The numerical data are presented as means of duplicate experiments (n = 3)

A number of apoptotic proteins, including Bcl-2, catalase, clusterin, HO-1, livin, XIAP, HSP70, Bcl-x, Bax and HSP60 were differentially expressed in the treated and untreated MDA-MB-231 cells (Fig. 6).

The expression levels of Bcl-2 protein were found to be significantly reduced in the combination 1-treated cells compared with the bromelain- (*P* = 0.002) and untreated cells (*P* = 0.001). The expression level of Livin, Bax, Catalase, HO-1 and XIAP were also found to be significantly reduced in the combination 1-treated cells compared with the bromelain- (*P* = 0.000) and untreated cells (*P* = 0.000). However, the expression levels of several anti-apoptotic proteins, including HSP70 increased significantly in the MDA-MB-231 cells treated with combination 1 compared with the bromelain- (*P* = 0.016) and cisplatin-treated cells (*P* = 0.001). Moreover, the expression level of Bcl-x were also found to significantly higher in the combination 1-treated cells compared with the bromelain- (*P* = 0.002) and cisplatin-treated cells (*P* = 0.000).The expression levels of pro-apoptotic proteins HSP60 were found to be significantly reduced in the cells treated with combination 1 compared with the bromelain- (*P* = 0.041) and untreated cells (*P* = 0.014).

**Fig. 7:**
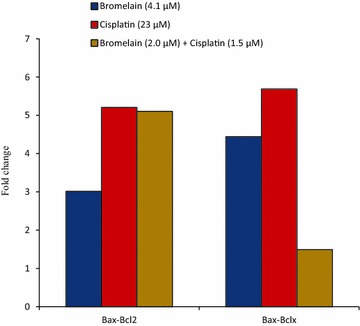
Fold-change values for Bax to Bcl-x and Bax to Bcl-2 in treated cells at 48 h. The numerical data are presented as mean ± SD (n = 3)

These results indicated that combination 1 affected the expression levels of most of the apoptotic proteins in MDA-MB-231 to a much greater extent than single-agent treatment with bromelain (4.1 µM) or cisplatin (23 µM). These results were therefore consistent with those of the JC-1 staining assay, with the fold-change values for Bax to Bcl-x and Bax to Bcl-2 suggesting the involvement of the mitochondrial pathway in the apoptosis induced by bromelain (4.1 µM), cisplatin (23 µM) and combination 1. The ratios of Bax to Bcl-2 and Bax to Bcl-x were calculated and the results are shown in Fig. 7. These results revealed that was an increase in the Bax/Bcl-2 ratio following the treatment of the cells with combination 1, which was accompanied by a decrease in the Bax/Bcl-x ratio.

**Fig. 8:**
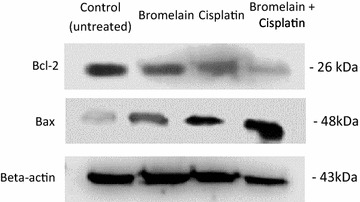
Combination 1 (bromelain (2.0 µM) + cisplatin (1.5 µM)) decreased Bcl-2 protein expression and upregulated Bax protein expression. Shown here are the western blot gel images for Bcl-2, Bax and Beta-actin 35 at 48 h

To validate the results obtained using the Proteome Profiler Human Apoptosis Array kit, we also examined the protein extracts obtained from the MDA-MB231 cells at 48 h by western blot analysis using anti-Beta-actin, anti-Bcl-2 and anti-Bax (Fig. 8). The results of this analysis showed that the MDA-MB-231 cells treated with combination 1 contained much lower levels of the anti-apoptotic protein Bcl-2 compared with the untreated cells (*P* = 0.000), as well as the bromelain-treated (*P* = 0.000) and cisplatin-treated cells (*P* = 0.000). By contrast, we observed a higher level of the pro-apoptotic protein Bax after 48 h following the treatment of the cells with combination 1 compared with the control cells (*P* = 0.009), bromelain-treated cells (*P* = 0.002) and cisplatin-treated cells (*P* = 0.043). The results of this western blot analysis therefore suggested that the treatment of the cells with combination 1 led to a much greater decrease in the expression of Bcl-2 compared with single-agent treatment with bromelain or cisplatin. Furthermore, the observed increase in the expression of Bax highlights the importance of the role played by the mitochondrial pathway in the apoptosis of the MDA-MB-231 cells triggered by combination 1.

## Discussion

It was envisaged that the combination of bromelain and cisplatin would lead to synergistic effects, which would enhance their anti-cancer effects towards MDA-MB-231 human breast cancer cells. The CI values and isobolograms obtained using the MTT data clearly showed that the treatment of the MDA-MB-231 cells with a combination of bromelain and cisplatin resulted in a synergistic and dose-dependent increase in the inhibitory activity for combination 1 (bromelain 2 µM + cisplatin 1.5 µM) towards the growth of these cells. We also demonstrated that the IC50 dose of cisplatin was reduced almost 13-fold in combination 1. Cisplatin is a well-known anti-cancer agent, which is used in clinical practice to treat a variety of different cancers. However, despite its effectiveness and broad range of activity, the use of cisplatin is associated with several adverse side effects [3, 4, 7–9]. In contrast, the cysteine endopeptidase bromelain is a non-toxic agent with proven anti-cancer activity against various cancers [17, 24]. The concentration ratio of bromelain to cisplatin played an important role in determining the extent of the synergistic effects, as exemplified by the differences observed for different concentrations of cisplatin and bromelain in the different combinations evaluated in this study.

Annexin V-FITC and cell cycle analyses were performed on the treated and untreated MDA-MB-231 cells to determine whether the growth inhibitory effects of combination 1 could be attributed to apoptosis. The results of these assays showed that the synergistic effects of combination 1 were caused by an augmented apoptotic response. This effect could also be attributed to a different mode of action to those employed by cisplatin and bromelain. Cisplatin exerts its activity through the formation of intra- and inter-strand DNA adducts that damage the DNA and interferes with its normal functions [6, 26]. In contrast, the inhibitory effects of bromelain are dependent on its proteolytic activity, which allow it to remove certain cell surface molecules associated with cellular migration and adherence [27]. The proteolytic activity of bromelain has also been reported to promote apoptosis in a number of human cancer cells, particularly in breast cancer cells [18, 28].

Consistent with our results, previous studies have demonstrated the involvement of the mitochondrial pathway in the apoptosis induced by single-agent treatment with cisplatin or bromelain in cancer cells [28–31]. The treatment of the MDA-MB-231 cells with combination 1 resulted in a loss of mitochondrial membrane potential, which suggested that the mitochondrial pathway was involved in the apoptosis induced by combination 1.

The results obtained using the Proteome Profiler Human Apoptosis Array kit showed that combination 1 led to decreases in the expression levels of several anti-apoptotic proteins, including cIAP 1, Bcl-2, catalase, clusterin, HO-1, livin, XIAP, HSP27 and claspin. Notably, combination 1 led to considerable decreases in the expression levels of cIAP1, catalase, clusterin, HO-1, livin and XIAP compared with the cells treated with bromelain or cisplatin. Interestingly, we also observed pronounced increases in the expression levels of several anti-apoptotic proteins, including HSP70 and Bcl-x in the cells treated with combination 1 compared with the untreated cells. In contrast, we did not observe any increases in the regulation of any pro-apoptotic proteins (*e.g.*, Bax). We also observed an increase in the expression of the apoptotic protein HSP60.

Taken together with the observed decreases in the anti-apoptotic proteins Bcl-2 and HSP27, the increase observed in the pro-apoptotic protein Bax provided clear evidence that combination 1 induced mitochondria-mediated apoptosis. This result was consistent with previous studies, which established the important roles played by Bcl-2, HSP27 and Bax in the regulation of apoptosis activated through the mitochondrial pathway [32–38]. We also observed significant increases in the levels of the anti-apoptotic proteins HSP70 and Bcl-x in the cells treated with combination 1. Bcl-x and HSP70 play essential roles as anti-apoptotic proteins by preventing the release of mitochondrial apoptogenic factors such as cytochrome C and apoptosis inducing factor (AIF), and consequently inhibit apoptosis [36, 39]. The elevated levels of Bcl-x and HSP70 observed in this study would be insufficient to shut down the apoptotic process induced by combination 1 in MDA-MB-231 cells. The fold-change values shown in Fig. 6 for the pro-apoptotic protein Bax to the anti-apoptotic protein Bcl-x indicated that the increase in Bax was much greater than that of Bcl-x, suggesting that the pro-apoptotic effects of upregulated Bax would overwhelm the anti-apoptotic effects of Bcl-x. It is therefore possible that some other pathway or mechanism was being activated or inhibited by combination 1 and that this process was compensating for the interference in mitochondrial mediated-apoptosis and contributing to the synergistic effect of combination 1.

We also observed pronounced decreases in the levels of the anti-apoptotic proteins cIAP 1 and XIAP in the combination 1-treated cells. This change was attributed to an increase in the expression of HSP70. Although HSP70 is well known for its cytoprotective effects, previous studies [40, 41] have also shown that elevated levels of HSP70 may have led to an increased susceptibility to apoptosis. Elevated HSP70 levels would result in an increase in the inhibition of IkB kinase activity, which would inhibit the activity of the NF-kB pathway, leading to the inhibition of NF-kB-dependent anti-apoptotic gene induction [40–42]. Given that the expression levels of cIAP and XIAP are highly dependent on the NF-kB pathway, the inhibition of the IkB kinase by elevated levels of HSP70 would lead to a reduction in the expression levels of cIAP and XIAP. This change could therefore explain the considerable decrease observed in the levels of cIAP and XIAP in this study.

## Conclusion

Bromelain in combination with cisplatin synergistically enhanced the induction of apoptosis in MDA-MB-231 cells.
